# Quality of Nursing Care as Perceived by Patients Treated for Multiple Myeloma in Polish Oncology Units: A Cross-Sectional Study

**DOI:** 10.3390/cancers17010153

**Published:** 2025-01-06

**Authors:** Magdalena Kurek, Tomasz Tatara, Jakub Świtalski, Adam Fronczak, Magdalena Tatara, Anna Augustynowicz

**Affiliations:** 1 Department of Public Health, Faculty of Health Sciences, Medical University of Warsaw, 02-091 Warsaw, Poland; 2 Department of Health Economics and Medical Law, Faculty of Health Sciences, Medical University of Warsaw, 01-445 Warsaw, Poland; 3 Faculty of Medicine, University of Social Sciences, 90-113 Łódź, Poland; 4 School of Public Health, Centre of Postgraduate Medical Education of Warsaw, 01-826 Warsaw, Poland

**Keywords:** quality of health care, multiple myeloma, nursing care, autologous transplantation, chemotherapy

## Abstract

**Background/Objectives:** Patient satisfaction is one of the indicators of the quality of nursing care. The purpose of this study is to find out the level of satisfaction of patients with multiple myeloma with the quality of nursing care in oncology units. **Methods:** Data were obtained by a diagnostic survey method, using the Newcastle Nursing Satisfaction Scale. The survey was conducted among patients from four oncology departments in Poland on the day the patient was discharged or transferred to another unit. Participation in the study was voluntary and required patient consent. Patients were assured of the anonymity of their responses. **Results:** The study included 65 men and 75 women treated with chemotherapy and autologous hematopoietic stem cell transplant. Experiences and satisfaction with nursing care presented a level of 71.80 points and 74.46 points, respectively. The analysis showed no statistically significant differences between the groups in terms of treatment and gender. A statistically significant negative association was shown between age and nursing care experience score (r = −0.19; *p* = 0.024). Positive associations were shown between length of stay on the unit and rating of experience of nursing care (r = 0.23; *p* = 0.006) and satisfaction with nursing care (r = 0.26; *p* = 0.002). **Conclusions:** The experience and satisfaction with nursing care among patients treated for multiple myeloma in Polish oncology units is moderate. Efforts should be made to improve the quality of nursing care, especially taking into account the needs of the elderly.

## Simple Summary

Individuals with cancer, including multiple myeloma, often have significant nursing needs. The specific nature of their condition and the functional difficulties from treatment impact their overall quality of life. Therefore, it is crucial to provide the highest quality care, addressing needs such as pain relief, health status monitoring post-therapy, and proper nursing activities. Considering patients’ perspectives and tailoring care to their needs is also important. In our study, we checked the opinions of multiple myeloma patients from Poland about the quality of nursing care. The results obtained give a picture of the situation and may be helpful in formulating areas requiring improvement.

## 1. Introduction

Modern nursing emphasizes continuous learning and the need for ongoing education and development, with the primary role of nurses being to provide comprehensive care to patients. The aim of nursing development is to enhance nurses’ skills, competence, and knowledge. Currently, in the health sciences, there is significant interest in quality of life and the quality of nursing care. Undoubtedly, the quality of nursing care affects patients’ quality of life [1]. Nurses address patients’ needs across various aspects of life, including mental, physical, spiritual, and cultural domains. Establishing a strong relationship and cooperation with patients is a crucial element of care [2].

Nowadays, patients are considered active partners in their care. They have access to information and medical records and are responsible for their health and decisions. Their opinions can influence evidence-based nursing. One way to assess the quality of nursing care is through patients’ subjective evaluations. Surveys measuring patient satisfaction reflect the quality of care provided.

Several factors influence the development of nursing, including the rising number of cancer cases, an aging population, and lifestyle-related diseases, all of which increase the demand for nursing services. Unfortunately, nurses face challenges, such as low staffing levels, work overload, shift work, and lack of equipment. These issues can lead to reluctance to guarantee quality services, a decline in the quality of care and safety for both patients and staff, and the occurrence of adverse events [1,3,4]. Evaluating the quality of nursing care is essential for its continuous improvement to meet patients’ expectations.

Individuals with cancer, including multiple myeloma, often have significant nursing needs. The specific nature of their condition and the functional difficulties from treatment impact their overall quality of life [5,6]. Therefore, it is crucial to provide the highest quality care, addressing needs such as pain relief, health status monitoring post-therapy, and proper nursing activities [7]. Considering patients’ perspectives and tailoring care to their needs is also important [8].

This study aims to determine the satisfaction of patients diagnosed with multiple myeloma with the quality of nursing care in oncology units. This article is part of a series of publications devoted to the care of patients with multiple myeloma. During a review of the available literature, scientific studies were found regarding satisfaction with nursing care among oncology patients, conducted using various research tools. No studies on the above-mentioned topic were found among patients with multiple myeloma, which indicates the originality of the study.

## 2. Materials and Methods

### 2.1. Study Population and Data Collection

The study’s respondents were patients from oncology units who had been diagnosed with multiple myeloma. The criteria for patient inclusion were age over 18 years, a confirmed diagnosis of plasmocytic myeloma, a minimum of 2 nights spent in the unit, ability to read and write in Polish, and no disturbances of consciousness. The study was conducted on the day the patient was discharged or transferred to another unit. Participation in the study was voluntary and required patient consent. Patients were assured of the anonymity of their responses. The questionnaires, prepared in an unsigned envelope, were distributed and obtained by a person who was not a member of the therapeutic team. After completing the survey and sealing the envelope, the patient could put the envelope in the box at the ward or return it to the interviewer. Some patients required assistance in completing the questionnaires due to their health condition. Assistance in completing the questionnaire consisted of reading aloud the questions asked to the patient and marking the answers provided by the patient. The examiner did not suggest what answers the patient should select. Information was obtained only from the patient. The patient’s family did not participate in the study because suggestions from third parties could have influenced the final result.

The study took place from August 2020 to December 2021 in four oncology units in Poland, with prior consent from the institutions’ management.

### 2.2. Research Tool

The study utilized a diagnostic survey method employing the Newcastle Satisfaction with Nursing Scale (NSNS), which is used to assess experiences related to nursing care and patient satisfaction with this care [9,10]. This tool is divided into three parts. The first part pertains to the experience of nursing care, containing 26 items (15 positive and 11 negative) on a 7-point Likert scale, with response options ranging from “fully disagree” to “fully agree”. The second part addresses 19 aspects of nursing care, with responses provided on a 5-point Likert scale (ranging from “not at all satisfied” to “completely satisfied”). Scores on both scales can range from 0 to 100, where 100 represents the best possible nursing experience or complete satisfaction with all aspects of nursing care. The third part focuses on patient demographic characteristics and issues related to the evaluation of the hospital stay. It includes two questions (“How would you rate the nursing care you received in this unit?” and “Overall, how would you rate your stay in this unit?”) rated on a 7-point Likert scale, with responses ranging from “dreadful” to “excellent”. Consent to use the research tool was obtained from the Department of Anesthesia and Intensive Care Nursing, Poznan University of Medical Science, along with instructions for conducting the examination. This is the place where the Polish version of NSNS was developed for use in Polish conditions. This is an adaptation of the original scale developed by researchers from the University of Newcastle upon Tyne [9,10].

### 2.3. Statistical Analysis

The data obtained from the questionnaires were entered into a Microsoft Excel spreadsheet and then statistically analyzed using IBM SPSS Statistics 29. An analysis of basic descriptive statistics was conducted, along with the Shapiro–Wilk test for normality of data distribution, the Mann–Whitney test, the Student’s *t*-test for independent samples, Pearson’s r correlation analysis, and the Kruskal–Wallis test. The significance level was set at α = 0.05. To verify the internal consistency of the questionnaire scales, Cronbach’s Alpha coefficient was calculated. The parametric Pearson test was used to determine the correlation because the distribution of variables is slightly asymmetric (Sk = −0.50). There are sources that say that if skewness does not exceed an absolute value of 2, parametric tests can be used [11,12].

## 3. Results

### 3.1. Sample

Questionnaires were distributed to approximately 500 patients, with 140 correctly completed surveys returned. Among these, 65 were completed by men and 75 by women. The analysis included 82 patients treated with chemotherapy and 58 treated with autologous hematopoietic stem cell transplantation (autoHSCT). All patients stayed in ordinary hematology/oncology departments. Patients treated with chemotherapy stayed in a room with several people, while patients treated with autoHSCT stayed in a single room because each auto-transplantation procedure requires the organization of appropriate sanitary conditions. The average age was 66, with the youngest patient being 43 and the oldest 83. The average length of stay in the unit was 26 days, ranging from a minimum of 3 days to a maximum of 115 days. Only 13 patients had a specific nurse assigned to their care. Patient characteristics are shown in Table 1.

### 3.2. Preliminary Analyses

Reliability analysis of the used questionnaire according to Cronbach’s Alpha indicator is presented in Table 2. The “experiences of nursing care” scale had good, and the “satisfaction with nursing care” scale had excellent reliability.

In the initial step of the analysis, the distributions of the quantitative variables were examined. Basic descriptive statistics were calculated, and the Shapiro–Wilk test was used to assess the normality of the distribution. The results of this analysis are presented in Table 3.

The result of the Shapiro–Wilk test for satisfaction with nursing care was statistically significant, indicating that the distribution of this variable significantly deviated from a normal distribution. However, the skewness did not exceed an absolute value of 1, suggesting that the distribution was only slightly asymmetric. Therefore, it was reasonable to proceed with further analysis using parametric tests.

### 3.3. Main Analyses

The next step was to examine whether patients treated with autoHSCT for multiple myeloma differed from those treated with chemotherapy in terms of satisfaction with nursing care. Due to the statistically significant inequality of the compared groups, the Mann–Whitney test was employed. The results of this analysis are presented in Table 4. No statistically significant differences were found between the compared groups regarding nursing care evaluation. This indicates that patients, whether treated with chemotherapy or autoHSCT, rated their nursing care experience and satisfaction similarly.

Next, gender differences in nursing care evaluation were examined. Using Student’s *t*-test for independent samples, women and men were compared regarding their nursing care evaluation. The results of this analysis are presented in Table 5. There were no statistically significant differences between the groups in terms of nursing care evaluation. This indicates that patients, regardless of gender, rated their experience and satisfaction with nursing care similarly.

In the next step of the analysis, the relationship between the length of stay on the unit and the evaluation of nursing care was examined. For this purpose, Pearson’s r correlation analysis was conducted, and the results are presented in Table 6. The analysis revealed statistically significant positive correlations between the length of stay on the unit and the evaluation of the nursing care experience and satisfaction with nursing care. This indicates that, as the number of nights spent on the unit increased, the patients’ evaluation of nursing care in terms of experience and satisfaction also improved.

Next, it was examined whether patients with basic vocational, secondary, and higher education differed in their satisfaction with nursing care. Due to the statistically significant inequality of the groups compared, the Kruskal–Wallis test was employed. The results of this analysis are presented in Table 7. The analysis revealed no statistically significant differences between the groups in terms of nursing care evaluation. This indicates that, regardless of whether the patients had basic vocational, secondary, or higher education, they rated their nursing care experience and satisfaction at similar levels.

The final step in the analysis was to determine if there was a relationship between the age of the subjects and their evaluation of nursing care. For this purpose, Pearson’s r correlation analysis was conducted, and the results are presented in Table 8. The analysis revealed a statistically significant negative relationship between age and the evaluation of nursing care experience. This indicates that, as the age of the subjects increased, their evaluation of the nursing care experience decreased.

Most respondents rated the overall nursing care as very good (40.7%). None of the survey participants rated nursing care as poor or very poor. Similarly, most patients rated their overall stay in the unit as very good (40%), and no patient found their stay to be dreadful or very poor. Table 9 below shows the percentage range of patients’ satisfaction ratings with nursing care and their stay in the unit.

## 4. Discussion

Patient satisfaction is a key indicator of the quality of nursing care. With the evolution of nursing, the increasing incidence of oncological diseases, and the aging population in the modern world, there are growing demands on nurses, particularly in meeting the needs and expectations of patients. The quality of care is determined not only by patient opinions but also by the relationships within the treatment team, the organization of work, access to appropriate equipment, and team management. Various models of care exist in nursing, and selecting the right one is crucial. This forms the foundation of the nurse’s care plan, aiming to achieve the best possible outcomes for the patient while also helping to reduce costs and optimize the work of the nursing team.

Modern nursing emphasizes a holistic and individualized approach to patient care, particularly in oncology. A study by Kousoulou [13], involving 150 cancer patients in Cyprus, found a link between individualized approaches and the quality of nursing care, highlighting the need for more personalized care to ensure high-quality nursing care.

The results of this study indicate that elderly patients with multiple myeloma rate the quality of nursing care less favorably. This may be due to unmet needs in this patient group. A study by Kang [14], involving 110 plasma cell myeloma patients receiving chemotherapy in South Korea, indicated that deterioration in functional quality of life is age-related, which may be linked to inadequate quality of care. Nursing care for elderly cancer patients is complex, requiring knowledge from several scientific disciplines, such as oncology and geriatrics, and a focus on factors that determine quality of life. Geriatric oncology care largely relies on the concept of Comprehensive Geriatric Assessment (CGA). This tool helps estimate the optimal treatment plan by determining the patient’s medical, psychosocial, and functional capabilities [15]. It is also helpful for developing a nursing care plan, as geriatric assessment tools can identify patient-specific problems and implement specific interventions. An essential element in the care of elderly oncology patients is the role of the Advanced Practice Nurse (APN), tailored to meet the needs of the elderly during cancer treatment [16].

A review of available literature and medical databases revealed no studies focusing on the quality of nursing care among patients with multiple myeloma. The tool used in this study is employed to assess the quality of care in various hospital departments, such as surgery, cardiology, urology, and neurology [17,18,19,20]. An interesting observation was made in the Rodríguez-Herrera study [21], conducted in Mexico on 298 cancer patients, which tested the applicability of the NSNS scale among cancer patients. The authors concluded that the scale needs refinement for use among oncology patients treated on an outpatient basis, as the presence of cancer and time spent in the hospital unit can affect patient satisfaction. Further research is needed with the NSNS scale in different patient groups.

Issues related to satisfaction with nursing care were discussed in studies using tools other than NSNS. For example, the cross-sectional study Adam 2017, conducted among 596 patients in four European countries, i.e., Sweden, Greece, Finland and Cyprus, using the Oncology Patients’ Perceptions of the Quality of Nursing Care Scale (OPPQNCS) tool, indicates that the quality nursing care is high, which is also associated with better treatment results. The authors rightly point out that the organization of care affects the quality of nursing care. The Nordic countries score higher in the quality of care than Greece and Cyprus because they employ nurses specializing in oncology care, ensuring continuity of care, which is very important for oncology patients [22]. The results of the Abu Sharour 2022 study among 517 cancer patients in three Arab countries, using the Quality of Oncology Nursing Care Scale (QONCS), show a moderate quality of oncology nursing care. Results varied depending on treatment duration and marital status [23]. In turn, the Uña Cidón 2012 study shows that the satisfaction of nursing staff improves the safety and quality of care [24].

Patients’ subjective opinions are a valuable source of information regarding nursing services. Studying patient satisfaction with the quality of nursing care is crucial, especially among elderly oncology patients, who face numerous challenges due to cancer, comorbidities, and aging. To develop a care model specific to this patient group, further large-scale research is necessary. Meeting the needs of patients with multiple myeloma, especially older ones, because this group of patients requires the most attention, is a goal in increasing patient satisfaction and the quality of nursing care. Collecting and developing patients’ subjective opinions/problems and organizing specialized nursing care throughout the therapeutic process can improve the safety and satisfaction of both nurses and patients and improve the quality of nursing care. Another essential element in improving the quality of care is the constant education and specialization of nurses in the field in question.

Analyzing the answers in detail, patients most often believe, according to the experience scale, that nurses do not provide sufficient information when patients need it, there is a long waiting time for nurses when they are called, and nurses only irregularly checked whether the patient is okay. According to the satisfaction scale, patients are dissatisfied with the amount of time the nurses spend with the patient. The information obtained may be useful in improving the quality of nursing care for patients with multiple myeloma.

The limitation of the study is its cross-sectional nature, which limits the possibility of analyzing data at different time intervals and examining the impact of various factors determining the quality of nursing care. Another issue worth paying attention to is the relatively low response rate, which amounted to 28%. One of the reasons could be the extensive questionnaire, containing a large number of detailed questions. Completing it required attention, focus and time. This could have discouraged the respondent from providing answers, especially during therapy. Patients who were frail or in poor health were likely to be reluctant to participate in the study. Due to the fact that multiple myeloma is a rare disease that requires specific treatment, access to patients was difficult. The size of the sample and the unequal division of groups constitute an additional limitation of the study. Another important limitation of the study was the lack of comparison of patients treated with different chemotherapy drugs or comparison depending on the symptoms of the disease and physical condition. We also did not perform a physical examination of the patients. One of the reasons was the extensive questionnaire, which often resulted in patients’ reluctance to cooperate. An additional physical examination could result in patients opting out of completing the questionnaire, which would entail a reduction in the size of the study group. However, it is worth conducting further research in this area, on a larger scale.

## 5. Conclusions

Experience and satisfaction with nursing care among patients treated for multiple myeloma in oncology units is moderate. Most patients rate their experiences and satisfaction with nursing care positively, regardless of the type of treatment received or their gender. However, as patients’ age increases, the quality of nursing care rating decreases. This suggests a need for a different approach and intensified care activities for elderly individuals with plasma cell myeloma. It is necessary to develop a care model specific to this group of patients, taking into account the needs resulting from the multiple myeloma treatment process and the aging process. Additionally, as the length of time spent in the unit increases, patients’ ratings of satisfaction with nursing care, in terms of both experience and overall satisfaction, also improve. Patients rate the quality of nursing care similarly, regardless of their educational background. Overall, efforts should be made to improve the quality of nursing care, especially taking into account the needs of the elderly.

## Figures and Tables

**Table 1:** Characteristics of patients.

Variable		*n*	%
Gender	Male	65	46.4
	Female	75	53.6
Age	<40	0	0
	40–60	38	27.1
	>60	102	72.9
Education	Primary school	2	1.4
	Vocational school	42	30
	Secondary school	61	43.6
	University level	35	25
Length of stay (nights)	<10	27	19.3
	11–20	47	33.6
	21–30	34	24.3
	>30	32	22.3
Primary nurse	Yes	13	9.3
	No	112	80
	I don’t know	15	10.7

**Table 2:** Internal consistency of Newcastle Satisfaction with Nursing Scale.

Scale	Cronbach’s Alpha	Number of Items
Experiences of nursing care	0.869	26
Satisfaction with nursing care	0.962	19

**Table 3:** Basic descriptive statistics of the studied variables.

Scale	M	Me	SD	Sk	Kurt	Min	Max	W	*p*-Value
Experiences of nursing care	71.80	71.15	11.06	−0.17	−0.21	44.23	97.44	0.98	0.116
Satisfaction with nursing care	74.46	75.00	16.93	−0.50	−0.08	25.00	100.00	0.96	<0.001

M—mean score; Me—median; SD—standard deviation; Sk—skewness; Kurt—kurtosis; Min—minimum value; Max—maximum value; W—Shapiro–Wilk test result.

**Table 4:** Comparison of patients differentiated by treatment modality.

Scale	Chemotherapy (*n* = 82)			AutoHSCT (*n* = 58)			Z	*p*-Value	η^2^
	Average Weight	M	SD	Average Weight	M	SD			
Experiences of nursing care	67.42	71.19	11.62	74.85	72.68	10.25	−1.07	0.285	<0.01
Satisfaction with nursing care	67.57	73.40	17.08	74.65	75.98	16.76	−1.02	0.309	<0.01

*n*—sample size; M—mean score; SD—standard deviation; Z—test statistics value; η^2^—effect size index.

**Table 5:** Comparison of patients differentiated by gender.

Scale	Men (*n* = 65)		Women (*n* = 75)		t	*p*-Value	95% CI		η^2^
	M	SD	M	SD			LL	UL	
Experiences of nursing care	71.13	11.78	72.38	10.45	−0.67	0.507	−4.96	2.46	0.11
Satisfaction with nursing care	73.20	18.39	75.56	15.61	−0.82	0.412	−8.04	3.32	0.14

*n*—sample size; M—mean score; SD—standard deviation; t—value of the test statistic; CI—confidence interval for the difference between the means; LL and UL—lower and upper limits of the confidence interval; η^2^—effect size index.

**Table 6:** Correlation of duration of stay in the unit and the rating of nursing care.

Scale	Parameter	Number of Night in the Unit
Experiences of nursing care	Pearson’s r	0.23
	*p*-value	0.006
Satisfaction with nursing care	Pearson’s r	0.26
	*p*-value	0.002

**Table 7:** Comparison of patients differentiated by education, in terms of rating of nursing care.

Scale	Answer	Average Weight	M	SD	H(2)	*p*-Value	η^2^
Experiences of nursing care	Basic vocational (*n* = 42)	67.51	71.66	10.14	1.36	0.508	<0.01
	High school (*n* = 61)	66.98	70.91	11.12			
	Tertiary (*n* = 35)	76.29	73.37	12.16			
Satisfaction with nursing care	Basic vocational (*n* = 42)	68.14	74.59	15.60	0.89	0.642	<0.01
	High school (*n* = 61)	67.30	73.27	17.27			
	Tertiary (*n* = 35)	74.96	75.94	18.53			

*n*—sample size; M—median; SD—standard deviation; H—test statistics value; η^2^—effect size index.

**Table 8:** Correlation of age and the rating of nursing care.

Scale	Parameter	Age
Experiences of nursing care	Pearson’s r	−0.19
	*p*-value	0.024
Satisfaction with nursing care	Pearson’s r	−0.15
	*p*-value	0.071

**Table 9:** Total rating of nursing care.

Item	Answer	N	%
How would you rate the nursing care you received on this unit?	Dreadful	0	0
	Very poor	0	0
	Poor	1	0.7
	Fair	30	21
	Good	43	30.7
	Very good	57	40.7
	Excellent	9	6.4
Overall how would you rate your stay on this unit?	Dreadful	0	0
	Very poor	0	0
	Poor	1	0.7
	Fair	29	20.7
	Good	47	33.6
	Very good	56	40
	Excellent	7	5

## Data Availability

The datasets generated during and/or analysed during the current study are available from the corresponding author on reasonable request.
